# Fuzzy Controller Design Using Evolutionary Techniques for Twin Rotor MIMO System: A Comparative Study

**DOI:** 10.1155/2015/704301

**Published:** 2015-04-19

**Authors:** H. A. Hashim, M. A. Abido

**Affiliations:** ^1^System Engineering Department, King Fahd University of Petroleum and Minerals, Dhahran 31261, Saudi Arabia; ^2^Electrical Engineering Department, King Fahd University of Petroleum and Minerals, Dhahran 31261, Saudi Arabia

## Abstract

This paper presents a comparative study of fuzzy controller design for the twin rotor multi-input multioutput (MIMO) system (TRMS) considering most promising evolutionary techniques. These are gravitational search algorithm (GSA), particle swarm optimization (PSO), artificial bee colony (ABC), and differential evolution (DE). In this study, the gains of four fuzzy proportional derivative (PD) controllers for TRMS have been optimized using the considered techniques. The optimization techniques are developed to identify the optimal control parameters for system stability enhancement, to cancel high nonlinearities in the model, to reduce the coupling effect, and to drive TRMS pitch and yaw angles into the desired tracking trajectory efficiently and accurately. The most effective technique in terms of system response due to different disturbances has been investigated. In this work, it is observed that GSA is the most effective technique in terms of solution quality and convergence speed.

## 1. Introduction

In the recent few years, unmanned autonomous vehicles are needed for various applications including Twin Rotor MIMO system (TRMS) which has been studied under many engineering applications including control, modeling, and optimizations. TRMS is emulating the behavior of helicopter dynamics [1] and its main problem can be summarized in solving high nonlinearities in the system in order to provide the desired tracking performance with suitable control signal.

Real coded genetic algorithm, particle swarm, and radial basis neural network are used for TRMS parameter identification without any former knowledge [2–4]. TRMS has been examined with different controllers such as four PID controllers with genetic algorithm to tune PID gains [5], decoupling control using robust dead beat [6], model predictive control [7], and *H*_*∞*_ control for disturbance rejection [8]. All aforementioned controllers are examined under hovering positions and switching LQ controller is used to switch the controller between different operating points [9]. Hybrid fuzzy PID controller shows good tracking performance in comparison to PID controller [10, 11]. Sliding mode control has been proposed in [12, 13] where fuzzy control and adaptive rule techniques are used to cancel the system nonlinearities. Both techniques apply integral sliding mode for the vertical part with robust behavior against parameters variations and they showed good results. However, their limitations reflected lie in the control signal and design complexity. Generally, fuzzy logic control (FLC) has been developed as an intelligent control approach for various applications in the presence of uncertainties. Fuzzy has been implemented with fuzzy control for nonlinear systems with unknown dead zone [14, 15], for output feedback of nonlinear MIMO systems [15, 16], for uncertain systems [17], and for systems with random time delays [18]. Also, observer based on adaptive fuzzy has been implemented successfully in [19–21]. Decoupling FLC will be used in this work to control TRMS by removing the coupling effect in addition to providing the desired tracking performance.

Evolutionary algorithms are important optimization tools in engineering applications and they are gaining popularity among the researchers. Particle swarm optimization (PSO) has been proposed as efficient optimization algorithm [22]. PSO has been successfully implemented in different engineering applications including identifying the path following footstep of humanoid robot [23], setting the control parameters for automatic voltage regulator [24, 25], and designing fuzzy PSO controller for navigating unknown environments [26]. Differential evolution (DE) was formulated as impressive evolutionary algorithm in [27, 28]. DE was successfully tested for various applications involving tuning multivariable PI and PID controllers of the binary Wood-Berry distillation column [29], optimizing delayed states of Kalman filter for induction motor [30] and optimizing the controller parameters of adaptive neural fuzzy network for nonlinear system [31]. A new optimization technique based on bees swarming was developed [32] and later artificial bee colony (ABC) emerged in [33]. ABC shows great results for many applications, for instance, employing ABC to find the optimal distributed generation factors for minimizing power losses in an electric network [34], defining the path planning and minimizing the consumption energy for wireless sensor networks [35]. Finally gravitational search algorithm (GSA) was proposed recently as promising evolutionary algorithm and shows impressive results [36]. GSA has been successfully implemented in many areas including fuzzy controller design [37, 38] and solving multiobjective power system optimization problems [39, 40].

In this work, the main contribution is proposing a decoupling PD fuzzy control scheme for the nonlinear TRMS. Controller parameters will be defined based on an optimization technique. GSA, PSO, ABC, and DE have been implemented for a comparative study in order to optimize the gains of a proposed controller for the nonlinear TRMS. Another contribution of this work is defining the minimum objective function in addition to finding the most robust technique with different initial populations. These optimization techniques will be used to tune PD gains and coupling coefficients. The proposed approach is investigated for TRMS at different operating conditions taking into account the need for cancelling strong coupling between two rotors and the specific range of control signals, and finally providing the desired tracking response. Generally, the results show the effectiveness of the considered techniques. The best performance was observed with GSA in terms of convergence rate and solution optimality. The paper is organized as follows.  Section 2 includes the problem formulation. The proposed control strategy is presented in Section 3. Optimization techniques will be discussed in Section 4. In Section 5, simulation results are presented and discussed and the effectiveness of the proposed approach is demonstrated. Finally, Section 6 concludes the main findings and observations with recommended future work.

## 2. Twin Rotor MIMO System Modeling

**Figure 1:**
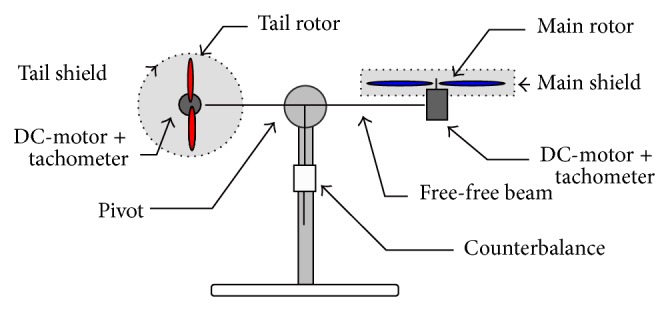
TRMS setup.

Twin rotor is a laboratory setup for stimulating helicopter in terms of high nonlinear dynamics with strong coupling between two rotors and training various control algorithms for angle orientations. The full description of TRMS has been detailed in [1], where the system has six states defined as *x* = [*x*_1_, *x*_2_, *x*_3_, *x*_4_, *x*_5_, *x*_6_]^*T*^, two control signals *u*_1_ and *u*_2_, and finally the output represented by *y* = [*x*_1_, *x*_3_]^*T*^. The main structure of TRMS studied in this work is shown in Figure 1.

The complete model of the system can be represented as follows:

(1) ddtx1=x2,ddtx2=a1I1x52+b1I1x5−MgI1sinx1 −B1ψI1x2+0.0326I1sin2x1x42 −KgyI1·a1x52+b1x5x4cos⁡x1,ddtx3=x4,ddtx4=a2I2x62+b2I2x6−B1φI2x4−1.75kcI2a1x52+b1x5,ddtx5=−T10T11x5+k1T11u1,ddtx6=−T20T22x6+k2T22u2.

TRMS dynamics are defined by six states as vertical or main angle, yaw or horizontal angle, vertical velocity, yaw velocity, and two momentum torques, respectively. The parameters of TRMS can be defined as follows: *a*_1_, *b*_1_, *a*_2_, and *b*_2_ are constant parameters referring to the static behavior of the system, two moments of inertia for vertical and horizontal rotors are stated as *I*_1_ and *I*_2_, friction momentums are *B*_1*ψ*_, *B*_2*ψ*_, *B*_1*φ*_, and *B*_2*φ*_, gravity momentum is *M*_*g*_, gyroscopic momentum is *K*_*gy*_, other parameters that have to be defined for vertical rotor are *T*_11_, *T*_10_ and for horizontal rotor *T*_22_, *T*_20_, and finally vertical and horizontal rotor gains are *k*_1_ and *k*_2_.

The control signals are used to control angles orientations by two torque momentum equations. Strong coupling between two rotors in addition to high nonlinearities detailed in (1) ended to formulate the tracking control as an interesting problem to be investigated. The solution of the control problem will be developed using decoupling proportional derivative fuzzy logic controller (PDFLC).

## 3. Proposed Control Approach

Since last few decades, fuzzy logic control [41] has been used extensively as intelligent technique in many control applications. In this work, decoupling PDFLC is proposed to solve coupling effects and high nonlinearities in addition to providing soft and smooth tracking response. The proposed control should be able to maintain the control signal in the demand range.

### 3.1. Structure of the Proposed Controllers

The proposed decoupling PDFLC scheme is mainly composed of four fuzzy controllers stated as vertical, horizontal, vertical to horizontal, and horizontal to vertical controllers as *V*, *H*, *VH*, and *HV*, respectively. The vertical controller is designed for the main rotor and horizontal controller is designed for the tail rotor. *HV* and *VH* controllers are designed in order to cancel the coupling effect between two rotors represented by the bias in the tracking response.

**Figure 2:**
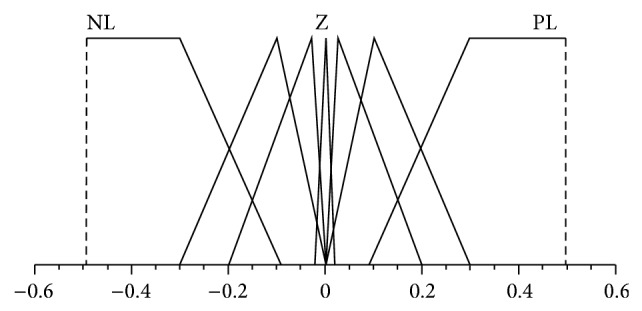
Membership fuctions of horizontal error and error rate.

**Figure 3:**
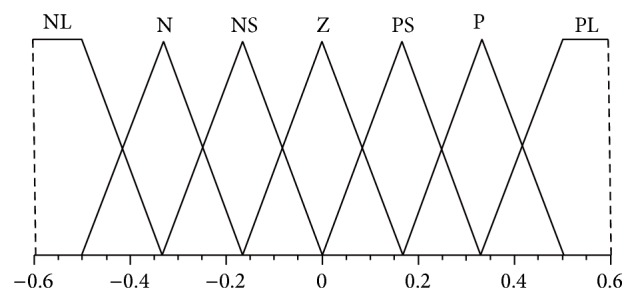
Membership fuctions of error and rate of vertical, vertical to horizontal, and horizontal to vertical fuzzy controllers.

**Figure 4:**
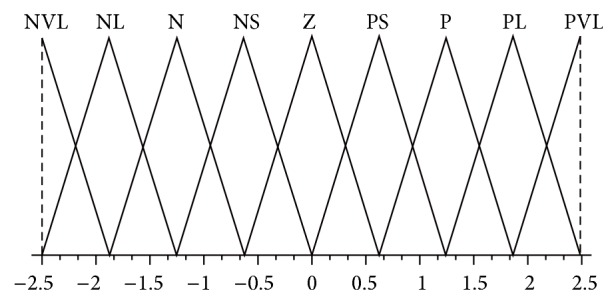
Membership functions of control signals of all fuzzy controllers.

The design of the assigned decoupling PDFLC for strong coupling and high nonlinear TRMS is shown in Figures 2, 3, and 4 as a triangular membership function. Inputs for PDFLC are expressed by error and rate of the error while the output is the control signals. The linguistic variables of the two input membership functions for the four PDFLC are described as PL, P, PS, Z, NS, N, and NL. The input of PDFLC ranged from −0.5 to 0.5 for the horizontal part and from −0.6 to 0.6 for the other three PDFLCs while output of the four membership functions is PVL, PL, P, PS, Z, NS, N, NL, and NVL within range −2.5 to 2.5. The linguistic variables are stated as PVL is positive very large, PL is positive large, P is positive, PS is positive small, Z is zero, NS is negative small, N is negative, NL is negative large, and NVL is negative very large.

**Figure 5:**
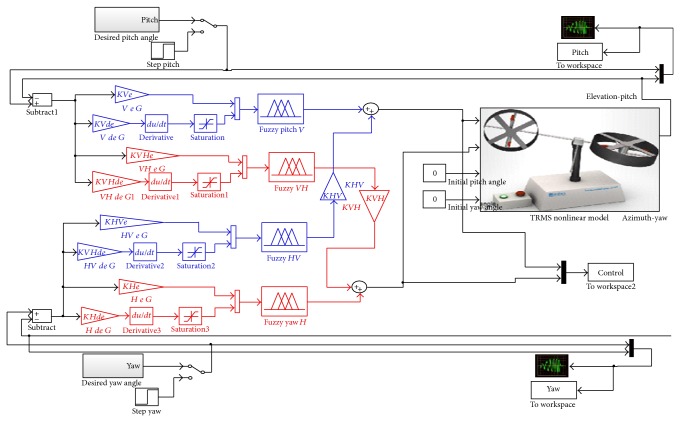
Proposed fuzzy controller for the nonlinear MIMO TRMS.

Table 1 describes the rule base of the proposed PDFLC.  Figure 5 shows the proposed controller of decoupling PDFLC. Ten gains will be tuned divided into eight gains for the proposed coupling PDFLC represented by four proportional gains and another four derivative gains in addition to two gains demonstrating the coupling effect from the output of HV and VH controllers.

### 3.2. Problem Formulation

Ten gains to be optimized are defined as *KVe*, *KVde*, *KHe*, *KHde*, *KVHe*, *KVHde*, *KHVe*, *KHVde*, *KHV*, and *KVH*, where *K* refers to gain, *V* refers to vertical, *H* refers to horizontal, *HV* refers horizontal to vertical, *VH* refers vertical to horizontal, *e* refers to error, and *de* refers to rate of error. The gains assigned to be between maximum and minimum constraints as follows:

(2) $$\begin{array}{c} 0.001\leq K_{\text{fuzzy}}\left(i\right)\leq 40\;\text{for}\;\;i=1,\ldots ,8.\\ -2\leq K_{\text{coupling}}\left(i\right)\leq 2\;\text{for}\;\;i=1,2, \end{array}$$

where

(3) Kfuzzy =KVe,KVde,KHe,KHde,KVHe,hhh KVHde,KHVe,KHVdeT,Kcoupling=KVH,KHVT.

The objective function is chosen to satisfy well-tracked response as follows:

(4) $$\begin{array}{c} \text{fit}=\overset{t_{\text{sim}}}{\underset{t=0}{\sum }}\left(e_{\psi }^{2}\left(t\right)+e_{\phi }^{2}\left(t\right)\right)\lambda \left(t\right), \end{array}$$

where

(5) $$\begin{array}{c} e_{\psi }\left(t\right)=\psi_{d}\left(t\right)-\psi \left(t\right),\\ e_{\phi }\left(t\right)=\phi_{d}\left(t\right)-\phi \left(t\right), \end{array}$$

*ψ*(*t*) and *ψ*_*d*_(*t*), are actual and desired vertical angles, respectively, *φ*(*t*) and *φ*_*d*_(*t*) are actual and desired horizontal angles, respectively, *e*_*ψ*_(*t*) and *e*_*φ*_(*t*) are errors between the desired and actual angles for vertical and horizontal parts respectively, and *λ*(*t*) is a weight factor in order to penalize the error as time increases. Ten gains will be optimized using four optimization techniques as mentioned in the literature. The objective function of each optimization technique is a minimization function considering gains have to satisfy the constraints in (2). In this study, GSA, PSO, ABC, and DE will be developed as a comparison study in order to search for the optimal gains.

## 4. Optimization Algorithms

This work presents a comparison study among four evolutionary optimization techniques. Each optimization algorithm aims to find the optimal gains for minimum possible objective function as defined in (4). The following subsections describe briefly optimization techniques implemented in this work.

### 4.1. Gravitational Search Algorithm

In the last few years, gravitational search algorithm (GSA) has been introduced as a new metaheuristic optimization algorithm developed by newton gravitational laws and was first proposed in 2009 by [36]. The algorithm stated that, for any two objects, every object is attracted to the other object by attraction force which is directly proportional to their mass and inversely proportional to their square distance. GSA has been explained in detail in [36].

**Figure 6:**
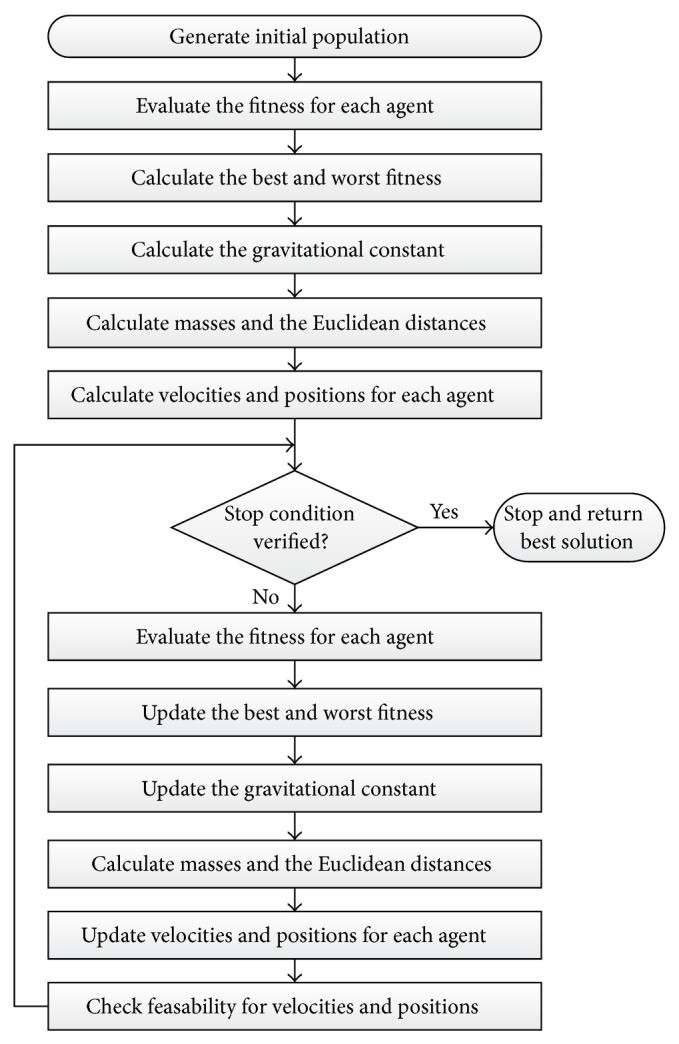
GSA computational flowchart.

GSA can be summarized in the following flowchart as shown in Figure 6.

### 4.2. Particle Swarm Optimization

Particle swarm optimization has emerged recently as combinational metaheuristic approach and was first inspired from a behavior combined between bird flocking and fish schooling in 1995 by [22]. PSO combines principles of human sociocognition in addition to evolutionary computation. Each particle in the swarm represents a potential or a solution which is required to be sought in the search space in order to find the optimal solution. A potential is formed by a set of agents. Two important equations are necessary to emulate socio and cognition behaviors are represented by position and velocity for each agent. The position of the agent can be defined by the following equation:

(6) $$\begin{array}{c} x_{i,j}\left(t\right)=v_{i,j}\left(t\right)+x_{i,j}\left(t-1\right). \end{array}$$

The velocity of each agent can be defined by

(7) vi,jt=αtvi,jt−1+c1r1xi,j∗t−1−xi,jt−1 +c2r2xi,j∗∗t−1−xi,jt−1,

where *i* = 1, 2,…, *N* and *N* is the population size, *j* = 1, 2,…, *m* and *m* are the size of agents in the potential, *x*_*i*,*j*_^∗^ is the local best solution, *x*_*i*,*j*_^∗∗^ is the global best solution, *α*(*t*) is a decreasing weight that can be defined by *α*(*t*) = exp⁡(−*α*(*t* − 1)*t*), *c*_1_ and *c*_2_ are positive constants, and *r*_1_ and *r*_2_ are uniformly distributed random numbers in [0,1]. PSO is described in detail in [22, 42].

**Figure 7:**
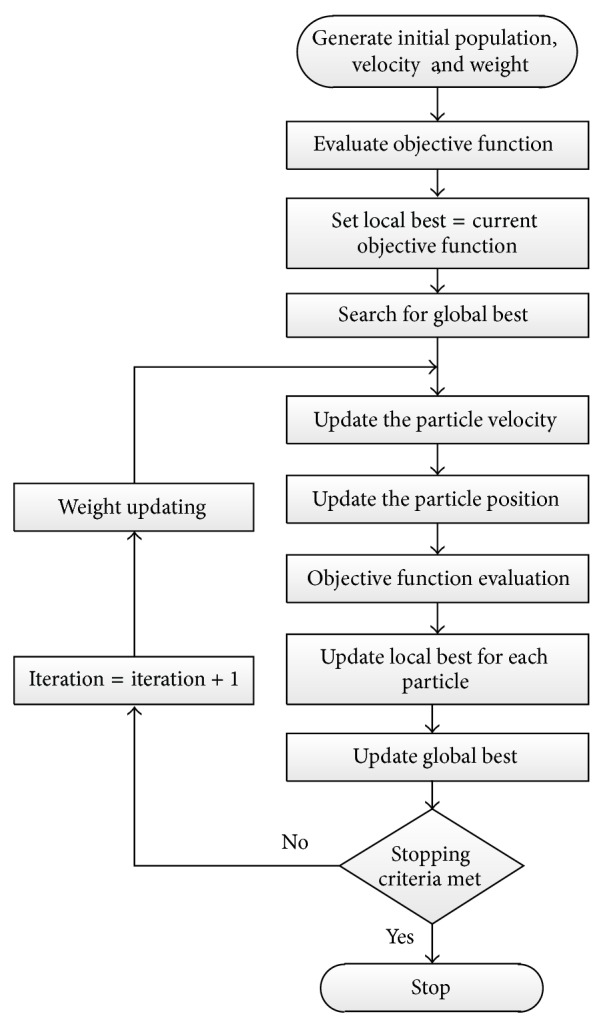
PSO computational flowchart.

PSO can be summarized in the following flowchart as shown in Figure 7.

### 4.3. Artificial Bees Colony

In the last few years, artificial bees colony has been introduced as a new metaheuristic optimization approach and was first inspired in 2005 by [32]. Colony of bees usually divided into three groups of bees as employed, onlooker, and scout bees. Life in bees' colony can be briefly summarized as employed bees search randomly for food where the best position of food is considered as the optimal solution. Employed bees dance to share information with other bees about amount of nectar and food source. Onlookers wait in the hive to receive information from employed bees. Onlooker bees can differentiate between the good source and the bad source and decide on the food quality based on dance length, dance type, and speed of shaking. Onlooker bees choose scout bees before sending them for a new process of food searching. According to food quality, onlooker and scout bees may decide to be employed and vice versa. The relation between bees food searching and ABC has been discussed in detail in [32, 33]. In the ABC algorithm employed and onlooker bees are responsible for searching in the space about the optimal solution while scout bees control the search process as mentioned in [33]. In ABC, the solution of the optimization problem is the position of the food source while the amount of nectar with respect to the quality refers to the objective function of the solution.

The position of the food source in the search space can be described as follows:

(8) $$\begin{array}{c} x_{ij}^{\text{new}}=x_{ij}^{\text{old}}+u\left(x_{ij}^{\text{old}}-x_{kj}\right). \end{array}$$

The probability of onlooker bees for choosing a food source is as follows:

(9) $$\begin{array}{c} p_{i}=\frac{{\text{fitness}}_{i}}{\sum_{i=1}^{E_{b}}{\text{fitness}}_{i}} \end{array}$$

with *i* = 1, 2,…, *E*_*b*_ and *E*_*b*_ is the half of the colony size, *j* = 1, 2,…, *D*, and *j* is the number of positions with *D* dimension, where *D* refers to number of parameters to be defined, fitness_*i*_ is the fitness function, *k* is a random number, where *k* ∈ (1, 2,…, *E*_*b*_), and *u* is random number between 0 and 1.

**Figure 8:**
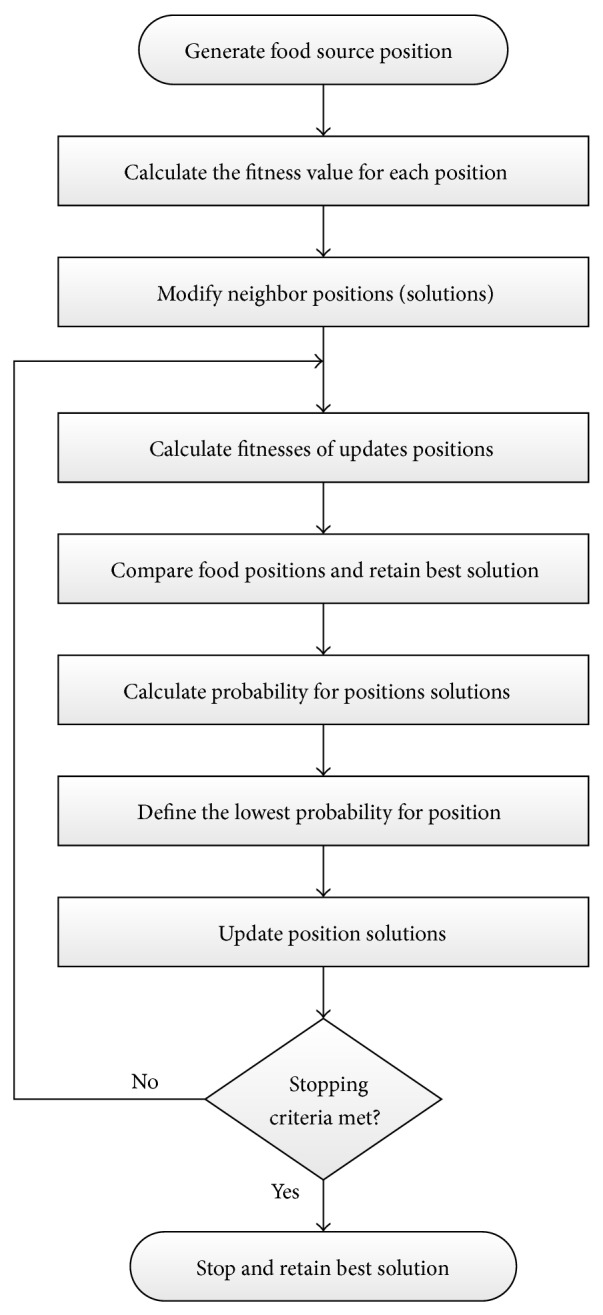
ABC computational flowchart.

ABC can be summarized in the following flowchart as shown in Figure 8.

### 4.4. Differential Evolution

Differential evolution has been developed as an optimization technique and has been tested on “Chebyshev Polynomial fitting problem” before adding several improvements [27]. Finally, DE has been formulated as impressive optimization technique in [28]. DE has the same structure of Genetic algorithm represented by crossover and mutation in addition to retaining the better population and best solution by comparing the old population with the new one. Important relations will be used in the searching process represented by mutation and crossover. Performing mutation requires assigning mutation probability (MP) arbitrarily as a constant number between 0 and 1. Mutation relation will be calculated only if MP is greater than a random number between 0 and 1 as follows:

(10) ViG+1 =XiG  +FXbestG−XiG+FXr1G−Xr2G.

The crossover will be computed by simple relation where crossover probability (CP) will be set arbitrarily between 0 and 1 and then it will be compared to random number between 0 and 1. The crossover step will be executed only if CP is greater than the random number. Crossover equation can be calculated from the following relation:

(11) $$\begin{array}{c} X_{i}\left(G+1\right)=V_{i}\left(G+1\right), \end{array}$$

where *i* = 1,2,…, *N*_*p*_ and *i* is iterated number for every solution in the generation, *X*_*i*_(*G*) represents a solution at iteration *i* in the generation, *V*_*i*_(*G* + 1) is a mutant vector generated from (10), *X*_*r*_1__(*G*), *X*_*r*_2__(*G*) are solution vectors selected randomly from current generation,*X*_best_(*G*) is the best achieving solution, and *F* is a random number between 0 and 1. DE is described in detail in [43].

**Figure 9:**
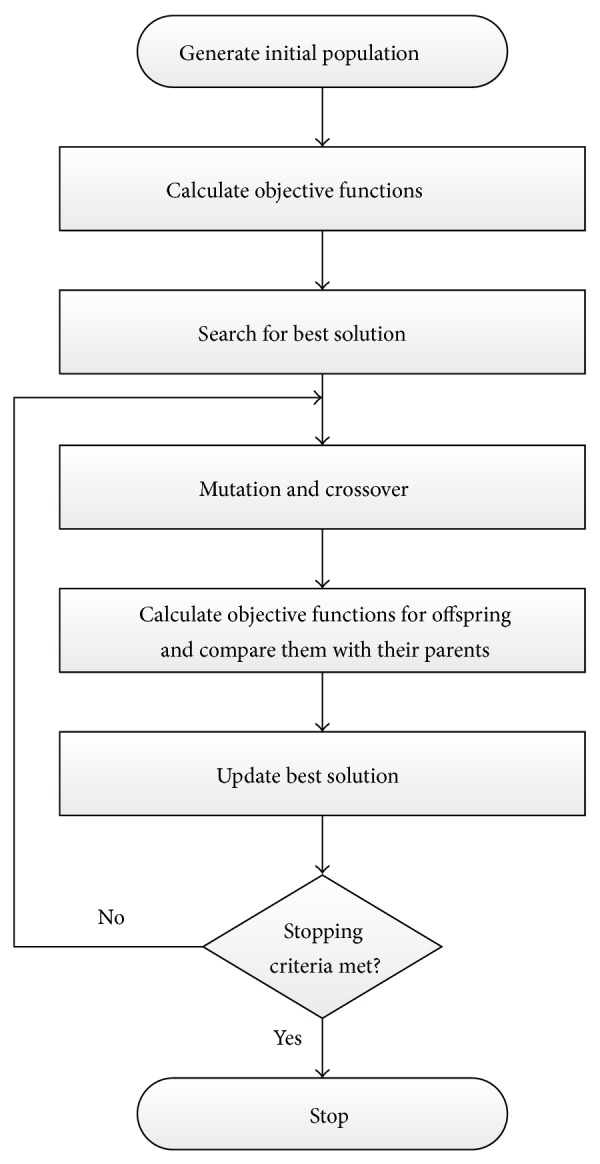
DE computational flowchart.

DE can be summarized in the following flowchart as shown in Figure 9.

### 4.5. Optimization Algorithms Implementation

For fair comparison, the population size is set as 150 particles for all techniques. For each particle, 10 parameters are defined to be optimized controller gains as shown in Figure 5. Initial settings for optimizations techniques are demonstrated in Tables 2, 3, and 4 for GSA, PSO, and DE, respectively, with setting maximum number of generations being 200.

## 5. Results and Discussions

Nonlinear TRMS has been simulated considering TRMS parameters in The appendix. Briefly, the system has been simulated for 80 seconds with initial conditions for both pitch and yaw angles are 0.1 and 0.15 rad, respectively, with 0.01 seconds sampling time. The objective function is computed from (4) where *λ*(*t*) is a penalty factor. To improve the settling time, the objective function will be multiplied by an increasing time weighting *λ*(*t*) which starts initially as *λ*(*t*) = 1. In this experiment, the reference has been chosen for both yaw and pitch angles to be 0.3sin(0.031*t*).

GSA, PSO, ABC, and DE are functioned to search for minimum error for 80 iterations in a number of experiments with different initializations.  Table 5 demonstrates the minimum error after 80 iterations of each experiment and their average values with their consumption time per iteration and also the number of setting parameters is discussed. It is noticed from Table 5 that GSA has the smallest average followed by DE then PSO and the highest average is ABC although GSA has more setting parameters than other comparison techniques.

**Figure 10:**
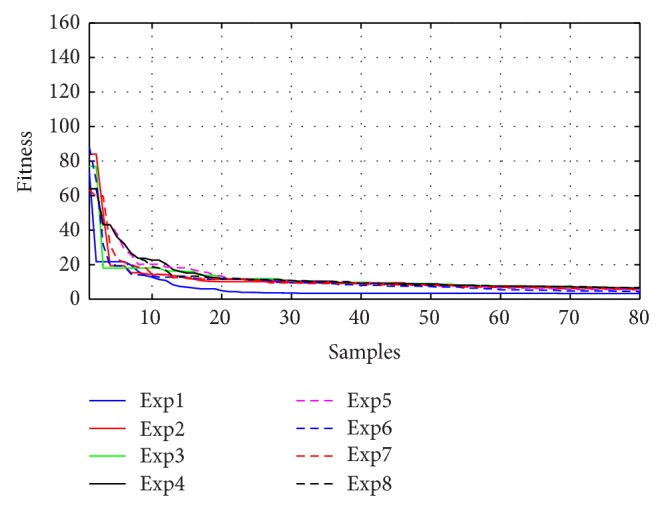
Fitness minimization for GSA with different initializations.

**Figure 11:**
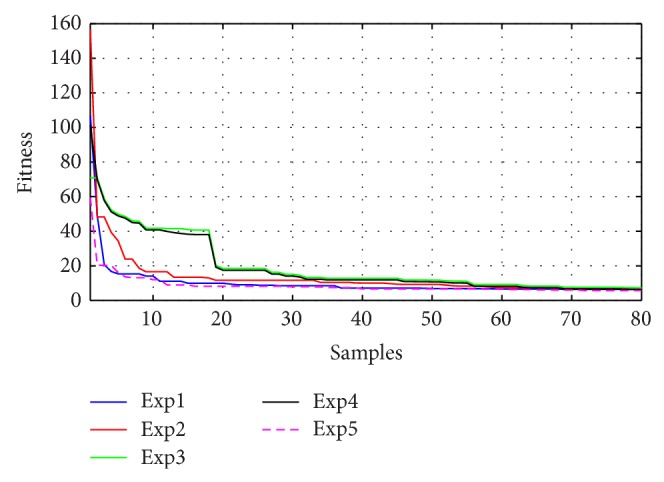
Fitness minimization for PSO with different initializations.

**Figure 12:**
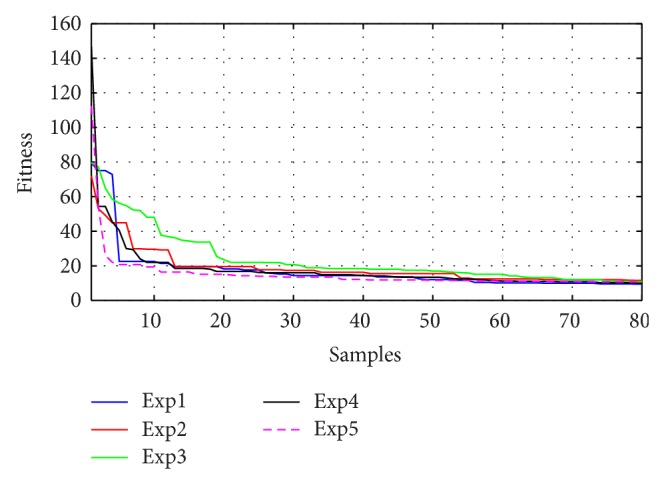
Fitness minimization for ABC with different initializations.

**Figure 13:**
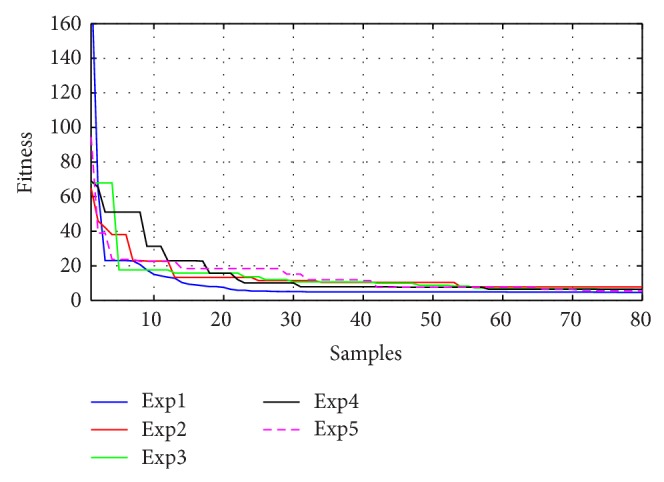
Fitness minimization for DE with different initializations.

Figures 10–13 present the fitness reduction for GSA, PSO, ABC, and DE, respectively, in 80 iterations. With different initial populations, GSA has been simulated in eight experiments while PSO, ABC, and DE have been simulated in five experiments in order to validate the robustness of the four search techniques.

**Figure 14:**
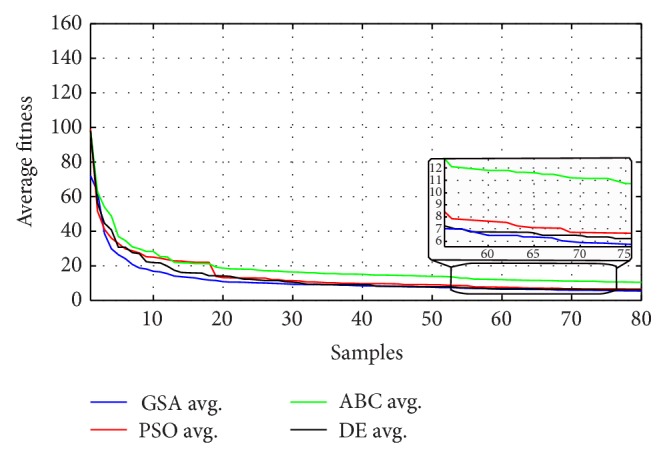
Average fitness for GSA, PSO, ABC, and DE.

The robustness for each method has been validated as shown in Figures 10–13 and Table 5 where the objective functions for each algorithm are very close by the end of 80 iterations.  Figure 14 demonstrates the average fitness function for each algorithm.

The optimal gains of each search technique with their minimum objective function after 200 iterations are expressed in Table 6. After 200 iterations and among the four comparison techniques, GSA gives the minimum error. On contrary, ABC gives the highest error.

In order to validate the presented results in Table 6, two different scenarios discuss the proposed technique where the first case is nonzero initial condition with sinusoidal input and the second case is zero initial condition with sinusoidal transient response.

**Case 1 .**

**Figure 15:**
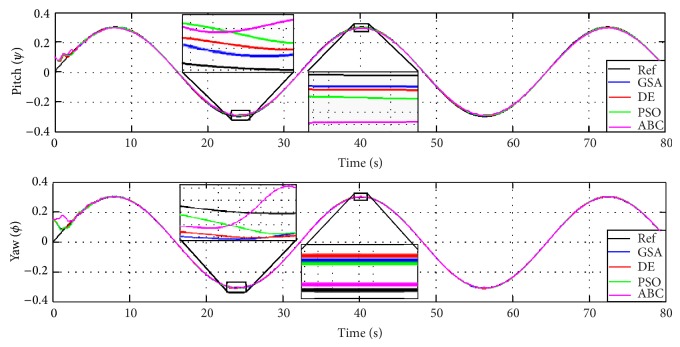
The proposed decoupling PDFLC controller response with GSA, PSO, ABC, and DE in Case 1.

Figure 15 shows the system response of the proposed fuzzy controller with initial conditions 0.1 and 0.15 for pitch and yaw angles, respectively. The reference input applied in this case is assigned to be 0.3sin(0.031*t*) for both pitch and yaw angles. The output response shows that the error is almost zero which demonstrates the effectiveness of the proposed controllers. Focusing on the tracking response, GSA shows better tracking performance and closer to the reference signal followed by DE while ABC shows the farthest in addition to some ripples at the peak point.

**Case 2 .**

**Figure 16:**
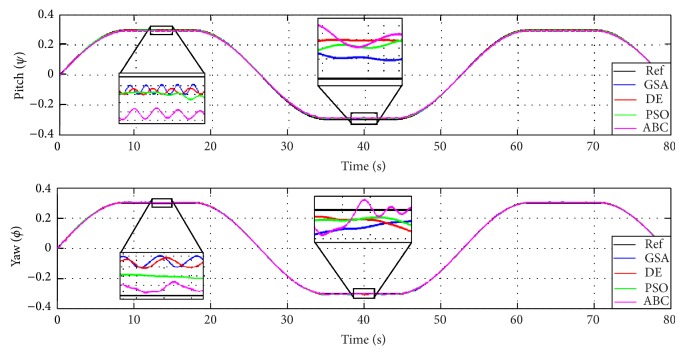
The proposed decoupling PDFLC controller response with GSA, PSO, ABC, and DE in Case 2.

In this case, Figure 16 has square wave reference inputs with soft transients for both angles where the frequency is 0.023 Hz. The output response shows good tracking results. Similar to Case 1, GSA shows close and well-tracked performance to the reference signal followed by DE in contrast to presence of ripples in ABC and a bit far from the reference input.

These two cases conclude that GSA is more robust and faster evolutionary algorithm in the search space than other three algorithms. Although four search algorithms give good tracking results with the proposed controller PDFLC, GSA is the most impressive technique with minimum objective function.

## 6. Conclusion

In this work, a comprehensive comparative study of four optimization techniques with decoupling PDFLC for high nonlinear TRMS has been proposed in order to cancel high nonlinearities and to solve high coupling effects in addition to maintaining the control signal within a suitable range. GSA, PSO, ABC, and DE have been implemented to tune the controller parameters and they showed great results in terms of tracking and error minimization. Robustness has been validated successfully for each technique with different initializations, optimizing the control parameters attempted by the optimization algorithms with two different operating conditions to test the efficacy of each algorithm. Finally, GSA shows the most impressive results in contrast to other algorithms with respect to convergence speed and optimum objective function. Implementing gain-scheduling technique with the decoupling PD fuzzy controller can be considered as a recommended future work.

## Figures and Tables

**Table 1:** Rule base of all fuzzy controllers.

Δ*e*∖*e*	NL	NM	NS	Z	PS	PM	PL
NL	NVL	NVL	NL	NM	NS	NS	Z
N	NVL	NL	NM	NM	NS	Z	PS
NS	NL	NM	NS	NS	Z	PS	PM
Z	NM	NS	NS	Z	PS	PS	PM
PS	NM	NS	Z	PS	PS	PM	PL
P	NS	Z	PS	PM	PM	PL	PVL
PL	Z	PS	PS	PM	PL	PVL	PVL

**Table 2:** Parameters setting for GSA.

Parameter	*α*	*λ*	ε	*G*_0_	*K*_best_

Setting	7	6	0.00001	1000	4

**Table 3:** Parameters setting for PSO.

Parameter	*λ*	*α*	*c*_1_	*c*_2_

Setting	10	0.99	2	2

**Table 4:** Parameters setting for DE.

Parameter	MP	CP	*F*

Setting	0.9	0.9	0.5

**Table 5:** Minimum error after 80 iterations and time per iteration.

	Exp1	Exp2	Exp3	Exp4	Exp5	Exp6	Exp7	Exp8	Average	Time per iteration (sec)	Setting parameters
GSA	3.2915	5.7112	5.8316	6.4720	5.4030	4.3753	5.7497	6.7643	5.4498	6383.15	5
PSO	6.3411	6.9329	7.2922	6.293	5.6631	—	—	—	6.5045	6382.10	4
ABC	9.4855	11.5355	10.7622	9.9329	10.7004	—	—	—	10.4833	6382.60	—
DE	4.6437	7.8255	6.3855	6.4262	5.4983	—	—	—	6.1558	6381.44	3

**Table 6:** Optimal gains after 200 iterations with their objective function.

	*KVe *	*KVde *	*KHVe *	*KHVde *	*KVHe *	*KVHde *	*KHe *	*KHde *	*KHV *	*KVH *	*Obj *
GSA	40	26.544	40	29.2786	1.7778	20.8239	7.3525	2.2567	−1.0862	−0.6442	3.0380
PSO	39.95	21.117	39.8855	17.201	1.3643	22.7434	7.3525	13.9838	−1.1284	−1.0432	3.9698
ABC	35.5195	19.1465	25.3081	3.0515	4.3111	24.4751	19.5009	16.1338	−1.2142	−1.0412	7.5166
DE	40	26.4236	40	32.5652	1.2728	20.0212	4.3722	3.5919	−1.0381	−0.8021	3.2915

**Table 7:** TRMS parameters.

Parameters description	Parameter value	Parameters description	Parameter value
*I*_1_ kg·m^2^	6.8 × 10^−2^	*a*_1_	0.0135
*I*_2_ kg·m^2^	2 × 10^−2^	*b*_1_	0.0924
*B*_1ψ_ (N·m·sec/rad)	6 × 10^−3^	*a*_2_	0.02
*B*_2ψ_ (N·m·sec/rad)	1 × 10^−3^	*b*_2_	0.09
*B*_1φ_ (N·m·sec/rad)	0.1	*k*_1_	1.1
*B*_2φ_ (N·m·sec/rad)	0.01	*k*_2_	0.8
*K*_*gy*_ (rad/sec)	0.5	*T*_11_	1.1
*M*_*g*_ (N·m)	0.32	*T*_10_	1
*T*_22_	1	*T*_20_	1

## Appendix

The parameters of the twin rotor MIMO system used in this study are given as shown in Table 7.
